# Consequences in Infants That Were Intrauterine Growth Restricted

**DOI:** 10.1155/2011/364381

**Published:** 2011-03-20

**Authors:** Erich Cosmi, Tiziana Fanelli, Silvia Visentin, Daniele Trevisanuto, Vincenzo Zanardo

**Affiliations:** ^1^Department of Gynecological Science and Human Reproduction, Maternal Fetal Medicine Unit, School of Medicine, University of Padua, Padua 35128, Italy; ^2^Department of Pediatrics, School of Medicine, University of Padua, Padua 35128, Italy

## Abstract

Intrauterine growth restriction is a condition fetus does not reach its growth potential and associated with perinatal mobility and mortality. Intrauterine growth restriction is caused by placental insufficiency, which determines cardiovascular abnormalities in the fetus. This condition, moreover, should prompt intensive antenatal surveillance of the fetus as well as follow-up of infants that had intrauterine growth restriction as short and long-term sequele should be considered.

## 1. Introduction

Fetuses with impaired intrauterine growth resulting from placental insufficiency are at increased risk of adverse short- and long-term outcome [1]. In fact, intrauterine growth restriction (IUGR) is a major cause of perinatal mortality and morbidity, and it is associated with several health problems throughout life. Current perinatal management of IUGR fetuses is primarily aimed at deciding the optimal timing of delivery, followed by neonatal intensive intervention to achieve optimal growth rates in order to avoid adverse perinatal complications [2].

IUGR can be regarded as a failure of a fetus that suffered nutritional deprivation to reach its genetic growth potential [3]. This is principally a vascular disorder, which consists of impaired fetal growth and in fetal multivessel cardiovascular manifestations [1]. The fetal circulatory response to placental insufficiency includes redistribution of the arterial circulation with preferential distribution of cardiac output towards the left ventricle, which mainly maintains an adequate oxygen supply to both the brain and the hearth. With further fetal deterioration, cardiac dysfunction results in abnormal venous flow velocity profiles, including reverse flow in the ductus venosus during atrial contraction and atrial pulsations in the umbilical vein.

Moreover, fetal cardiac function differs in IUGR compared to AGA fetuses as placental insufficiency affects fetal hearth. In fact, IUGR fetuses show an altered cardiac function in both systolic and diastolic phase, which determine an earlier and more pronounced right than left and diastolic than systolic fetal cardiac function deterioration [4].

Fetuses are classified as IUGR if estimated fetal weight (EFW) is below the 10th percentile and umbilical artery pulsatility index (PI) > 2 SD.

## 2. Short-Term Outcome

Intrauterine growth restriction is of huge importance in obstetric practice. A modern classification system of stillbirth, ReCoDe, has shown that IUGR is the most common factor identified in stillborn babies. In addition, it has serious consequences for babies who survive. IUGR is associated with increased risk of premature birth; increased morbidity among premature neonates, including necrotizing enterocolitis; low Apgar score; hypoxic brain injury and its long-term sequelae; the need for respiratory support and chronic lung disease; retinopathy of prematurity; prolonged neonatal intensive care unit (NICU), and mortality [3]. Furthermore, a number of causes of IUGR are associated with an increased risk of IUGR and intrauterine death (IUD) in mother's subsequent pregnancy [5]. 

The perinatologist plays a fundamental role in diagnosing the presence and cause of IUGR in pregnancy to avoid major consequences, through ultrasound assessment of estimated fetal weight and Doppler examination of fetal circulation. In particular, it appears to be a link between the length of intrauterine deficit and perinatal outcome.

## 3. The Barker Hypothesis and Fetal Programming of Adult Disease

Low birth weight, caused either by preterm birth and/or intrauterine growth restriction, was recently known to be associated with increased rates of cardiovascular disease and noninsulin-dependent diabetes in adult life [6–10].

The “developmental origins of adult disease” hypothesis, often called “the Barker hypothesis” proposes that these diseases originate through adaptations of the fetus when it is undernourished. These adaptations may be cardiovascular, metabolic, or endocrine, and they may permanently change the structure and function of the body, increasing coronary heart disease risk factors, such as hypertension, type 2 diabetes mellitus, insulin resistance, and hyperlipidaemia [11–14]. This hypothesis originally involved from observation by Barker and colleagues that the regions in England with the highest rates of infant mortality in the early 20th century also had the highest rates of mortality from coronary heart disease decades later. As the most commonly registered cause of infant death at the start of 20th century was low birth weight, these observations led to the hypothesis that low-birth-weight babies who survived infancy and childhood might be at increased risk of coronary heart disease in later life [15].

As Barker and his colleagues reported in several epidemiological and anthropological studies, in fetal life, tissues and organs go through the so-called “critical” periods of development. These may coincide with periods of rapid cell division. Although the fetal growth is influenced by its genes, several studies suggest that it is usually limited by intrauterine environment, in particular the nutrients and oxygen received from the mother. Influence linked to fetal and placental growth have an important effect on the risk of coronary heart disease and stroke. Thus, this theory focusing on intrauterine life, offers a new point of departure for research in cardiovascular disease. According to the thrifty phenotype hypothesis, deficient fetal supply may be followed by a programming, which includes circulatory adjustment and insulin resistance in liver and muscular tissue in order to spare the brain [6–10, 16]. 

In addition, postnatal overnutrition following intrauterine growth restriction may be pathogenetic for the development of obesity and type 2 diabetes whereas the elevated cardiovascular mortality rate may be associated with rapid postnatal catch-up growth in early infancy [17]. Many reports of relationship between birth size and disadvantageous glucose and insulin metabolism have widely been reviewed. In particular, fetal growth retardation has been associated with increased insulin resistance, higher fasting insulin concentrations, and increased incidence of type 2 diabetes. Neonatal abdominal circumference has been shown to predict plasma cholesterol and fibrinogen levels in adults in later life, which are both risk factors for cardiovascular disease. 

Association between IUGR and raised blood pressure in childhood and adult life has been extensively demonstrated around the world. In 1996, a review based on 34 studies involving more than 66.000 persons of all ages identified a negative relationship between birth weight and systolic blood pressure in childhood and adulthood. This relationship was independent of body size at time of blood pressure measurement, and its magnitude tended to increase with age [18, 19]. 

A similar review in 2001 based on 27 independent observational studies also evidenced a mean difference in systolic blood pressure of −1.7 mmHg per kilogram increment in birth weight. The review also documented a consistently negative association between birth weight and diastolic blood pressure. Besides low birth weight, 3 other early factors that are considered to be important risk factors for developing high blood pressure in adult life have been identified in individuals with IUGR. First, accelerated postnatal growth in weight and length is suggested to increase the risk for developing hypertension and type 2 diabetes in later life. Second, it was postulated that altered angiotensin activity was an important factor underlying the “fetal origins of adult diseases” hypothesis. Also, hypoxia increased sympathetic nerve activity and catecholamine production and proliferation of juxtaglomerular cells (and thus renin-producing cells) are suggested as factors in the pathogenesis [20, 21]. A followup study recently published by Cosmi et al. demonstrates that systolic blood pressure measurements in 2-year-old children born intrauterine restricted are significantly higher compared to appropriate for gestational age children at the same age [22]. 

From a historical viewpoint, it is clear that the Barker hypothesis received significant support because of its public health implication. However, it must be considered that many confounders are known to influence blood pressure in adult life apart from birth weight. In addition, a larger genetic heterogeneity of individuals enrolled may explain its results. However, as stated by Gluckman and Hanson “it is no longer possible to ignore the developmental phase of life” [23] and followup studies in early childhood will assist the medical community and public health to designing interventions in order to ensure the best possible fetal development [19].

## 4. Endothelial Dysfunction

The endothelium controls vascular tone, coagulation, and inflammatory responses. Endothelial dysfunction is an early event of atherosclerosis, preceding structural changes in the vascular wall. Atherosclerosis is thought to begin in childhood and to develop silently before clinical events such as myocardial infarction or stroke occur [24]. 

As with major cardiovascular risk factors, impaired growth in utero is associated with functional (endothelial dysfunction) and structural (increased wall thickness) changes to the arterial vascular consistent with early atherosclerosis. Impaired fetal growth in infants is associated with increased sympathetic tone and lipid concentration, and reduced concentration of insulin-like growth factor-1, all of which might contribute to arterial wall thickening. However, these findings are difficult to interpret because of potential confounding by, or interaction with, postnatal influences, so this hypothesis needs further clarification. Teeninga et al. demonstrated atheromatous changes in the aorta of children. Atheromatous changes have been documented histopathologically by early childhood, and it has been assessed that the first atherosclerotic lesions actually begin to develop in the abdominal aorta [25]. Nowadays, many studies demonstrated atherosclerotic wall thickening in the arteries of children with cardiovascular risk factors using ultrasound imaging assessing noninvasively early vascular changes. 

In 2005, Skilton and coworkers added new interesting information to Barker's hypothesis. They compared intima-media thickness (aIMT) of the aortic wall in newborn infants with low birth weight with normal controls. In IUGR newborns aIMT was significantly greater than in the controls. On the basis of these finding, the ultrasound-based measurement of abdominal aortic intima-media thickness in children was described as a feasible, accurate, and sensitive marker of atherosclerosis risk, and, as there was no confounding from childhood and adult exposures, they provided clear indications for a fetal contribution to later cardiovascular disease [26]. Also, Koklu et al. in 2007, evaluated the potential use of aIMT in the study of high-risk neonates, concluding that aIMT measurement in IUGR newborns can help in the early identification of asymptomatic vascular dysfunction [27–29]. Recently, Cosmi et al. studied aIMT in fetuses for the first time and then in the same infants after a mean followup of 18 months. This study showed that aIMT measurements in IUGR fetuses were inversely related to estimated fetal weight, showing that low birth weight and Doppler abnormalities may be correlated to an altered vascular structure causing possible endothelial damage, consistent with the finding that atherosclerosis begins to develop first in the intima of the aorta. These differences were present also at the time of followup [30].

Similarly, carotid intima-media thickness has been shown to be greater in children with low-birth weight. In a recent study, Crispi et al. confirms the presence of increased carotid wall thickness in children with IUGR and that these changes persist into childhood. The increased arterial wall thickness could be the result of vascular remodeling linked to metabolic programming in intrauterine restricted life [31]. They also highlighted that children with IUGR show changes in cardiac morphology, subclinical cardiac longitudinal dysfunction, and hypertension, all of which increase linearly with the severity of growth restriction and are independent of gestational age at delivery, lipid profile, or body mass index. 

The importance of early identification and intervention in pediatric risk factors for cardiovascular disease is now well recognized, and this could open new opportunities for monitoring and intervention in newborns and children affected with intrauterine growth restriction.

## 5. Fetal Programming and Renal Consequences

According to the fetal programming, the kidneys too appear to be extremely susceptible to intrauterine growth restriction and are often found small in proportion to body weight [32]. Several studies in animals and humans have described a reduced number of nephrons after IUGR. This results in an inborn decreased glomerular filtration surface area while renal blood flow per glomerulus is increased in attempt to maintain a normal overall glomerular filtration rate. According to the hyperfiltration hypothesis explained by Brenner and coworkers [33–35] this leads to glomerular hypertension and hypertrophy, which causes systemic hypertension and higher sodium reabsorption and glomerular damage resulting in albuminuria and glomeruloclerosis. Also, premature birth implies a shorter period of active nephrogenesis, as describes by Rodríguez et al., involving in impaired renal development [36]. Therefore, IUGR can lead to impairment of renal function. A kidney with a reduced nephron number has less renal reserve to adapt to dietary excesses or to compensate for renal injury. The pathway leading from small kidney to hypertension may include the rennin-angiotensin system, which is altered in the early phase of primary hypertension. An increased activity of the rennin-angiotensin system could be a compensatory mechanism in a decreased number of nephrons in order to maintain normal filtration [37]. These mechanisms are well described in experimental study including IUGR male rats, whose presented higher blood pressure and fewer glomeruli at 22 weeks of age [38]. In the last five years, more clinical data are available regarding maturation of renal function in IUGR infants. Keijzer-Veen and colleagues in 2005, identified a positive association of birth weight and glomerular filtration rate (GFR). The investigators also detected a negative association of birth weight and serum creatinine, suggesting that IUGR individuals are at greater risk to develop hypertension and progressive renal failure [39]. This work made a significant contribution to understanding mechanism associated with the progression of cardiovascular disease and intrauterine retardation. In contrast Rakow et al. found that glomerular filtration rate and urinary protein patterns were similar between IUGR 12-year-old children and controls, but kidney volume was smaller in the first group [40]. A meta-analysis published by Teeninga et al. in 2008, consisted of 201 patients (25 SGA, 176 AGA), showed that low birth weight has a strong influence on glomerular filtration rate and proteinuria, associated with a higher chance of developing several complications, including hypertension [41]. A recent followup study demonstrated that 5-year- old children born IUGR showed higher blood pressure, increased albuminuria, lower GRF, and different urinary sodium excretion rate than controls. These observations support the contention that extrinsic renal injury is not a prerequisite for the initiation and perpetuation of renal injury and that certain circumstances, prenatally derived, intrinsic deficiencies in functioning renal mass may be sufficient to contribute to renal functional decline occurring with advancing age.

## 6. Infant Neurodevelopment

Intrauterine growth restriction plays a significant role in short- and is long-term outcome and reflected in high incidence of brain dysfunction and neurodevelopmental impairment, as well as in somatic growth failure. These clinical consequences could not be apparent until later in childhood development and could involve poor academic performance, memory, visuomotor, and language difficulties, and executive function problems. Several longitudinal studies have addressed the question of correlation between cognitive development and somatic growth in IUGR, using a different questionnaire dealing with aspects of reading, writing, spelling, and mathematics. An increased risk of cognitive impairment has been demonstrated in children with small head circumference [42]. Shenkin et al. found that birth weight is significantly related to IQ at age 11 [43]. In other studies, IUGR children have lower nonverbal and verbal IQ than controls [44]. According to data from the National Collaborative Perinatal Project (1959–1976) the IQ scores of 2719 IUGR children tested at age 7 were 6 points lower than AGA children. Visuomotor development was also lower in IUGR group. Besides neurosensory handicaps, also behavioral sequelae are of serious concern. Behavioral problems, which might manifest only at school age, can have a great impact on school performance and social competence and have a negative influence on quality of life [45]. Learning difficulties and behavioral problems are reported more often in IUGR preterm infants compared to AGA [46].

Management of these infants is controversial. They have an increased perinatal mortality and morbidity including behavioral problems, minor developmental delay, poor neurosensory development, and spastic cerebral palsy. Definition of these important long-term relationships invites research of pathological mechanism. Recent advanced magnetic resonance imaging studies have shown that IUGR is associated with structural differences that can be identified very early in life, such as reduction in cerebral cortical grey matter, hippocampal volume, and sulcation index. These macrostructural alterations have been associated with microstructural and metabolic changes [47]. As explained by Baschat et al. [48], the IUGR fetus “enables” sparing of the head while growing in a low-nutrient intrauterine environment and has neuroadaptative modification aimed at preserving brain oxygen supply in presence of chronic hypoxia. This process is identified clinically by a reduced Doppler pulsatility index (PI) in the middle cerebral artery (MCA) [49]. When multiple parameters are considered, such as gestational age and birth weigh, umbilical artery reversed end diastolic velocity (UA-REDV) is identified as an important contributor to neurodevelopment. No similar effect could be demonstrated for abnormal venous Doppler findings. Brain sparing and cerebral arteries Doppler results are objects of study as predictors of adverse neurological outcome, but these relationships are not well assessed [45, 50]. These findings have important implications for the prognosis and the management of intrauterine growth restricted fetuses and children, who should be closely followed up to identified individuals at risk [42].

## 7. Conclusions

The importance of IUGR in understanding adverse pregnancy outcome is relevant not only for clinicians, but also for public health service. During the last decade, knowledge of the short-term and long-term consequences of impaired fetal growth has increased at a very rapid rate and has involved lots of clinical aspects. At present, it is most important not only to develop efficient methods of preventing and diagnosing IUGR, recognizing at-risk fetuses, and screening fetal growth restriction among low-risk pregnancies, but to work out followup and adequate treatment programs for the control of the disorders which may follow this conditions. Programming the right time to deliver is the best method to avoid adverse perinatal outcome; indeed, proper postnatal feeding and infant growth may be essential for long-term outcome.
